# Sound solutions: expanding access through digital audiobooks in an academic health sciences library

**DOI:** 10.5195/jmla.2026.2425

**Published:** 2026-10-01

**Authors:** Julie Evener, Melinda H. Berg

**Affiliations:** 1 jevener1@usf.edu, Director for Content Management & Discovery, USF Health Libraries, University of South Florida, Tampa, FL; 2 mberg4@usf.edu, Cataloging & Metadata Librarian, Assistant Librarian, USF Health, University of South Florida, Tampa, FL

**Keywords:** Audiobooks, OverDrive, Libby, academic health sciences libraries

## Abstract

**Background:**

As digital audiobooks gain popularity among the general public, academic health sciences libraries have the opportunity to provide access to supplemental nonfiction titles in this format which while often found in public libraries has not been traditionally offered in this setting.

**Case Presentation:**

An academic health sciences library serving students and faculty in the colleges of medicine, nursing, pharmacy, and public health piloted a collection of nonfiction health sciences audiobooks through OverDrive's Libby app. We launched an initial collection of over 500 titles using the cost per circ (CPC) pricing model, including a focus on four content areas: popular nonfiction, first person experiences, history of healthcare, and study helps. Over the first year of the project, we saw 123 total checkouts of 64 titles across 74 unique users, spending $351.84 US on CPC checkouts.

**Conclusions:**

The OverDrive title library includes sufficient offerings for academic health sciences libraries to create targeted nonfiction audiobook collections. Challenges of which other libraries should be aware include planning budgets around adding funds each year and determining local workflows to keep the collection updated. Though usage was low in the first year, it was consistent and varied, making the project beneficial and sustainable.

## BACKGROUND

Though audiobooks have a long history (e.g., Talking Books and Books on Tape), it is only recently due to technological advancements that they are becoming a mainstream way to consume books. A 2023 survey of U.S. adults found that 53% percent of respondents reported ever having listened to an audiobook; the percentage had remained steady at 44–46% for the three years prior [1]. Audiobook sales revenue is also steadily growing, showing the increased interest in and usage of audiobooks among consumers. In 2018, audiobook sales revenue was $0.94 billion, increasing to $2.2 billion in 2024 [2]. Dali and Brochu shared that “readers with print disabilities are no longer the primary or the largest audience for audiobooks, and … the popularity of audiobooks has grown tremendously” [3].

Overall increased demand for audiobooks has included an increased demand for audiobook availability in libraries. Though public libraries have been leading this charge due to a focus on audiobooks for leisure reading [3], the value of audiobooks for academia, and thus academic libraries, is also worth exploring. One study found that audio recordings of course lectures helped undergraduate students improve their understanding of biochemistry topics [4]. Though some researchers have explored the question of the effect of reading versus listening on mental load and comprehension for individuals without learning disorders [5], no definitive answer has yet been discovered [6–8].

However, that some modern students prefer consuming information via audio, including audiobooks, has been established. For example, a recent study of health sciences students at a Florida university found that 97% had read for leisure within the last 6 months, including 45% of those via audiobook [9]. Within the same group of respondents, two-thirds were interested or very interested in the health sciences library offering health-related leisure or supplemental books for students. The age group most typically found on college campuses—18 to 29 year olds—was also the age group who reported the highest proclivity for listening to audiobooks. Of 18 to 29 year old respondents in a 2021 survey, 30% reported that they had listened to an audiobook within the last 23 months (versus 27% of 30–49 year olds, 22% of 50–64 year olds, and 12% of 65+) [2]. Students responding to a leisure reading survey at Sam Houston State University indicated that they used the public library to get audiobooks (22.9%), and 45% of respondents said they would like to see audiobooks available through the university library [10].

As students in higher education, including medical and health sciences students, become ever-busier, audiobooks could be a great way for them to squeeze reading—whether required course reading, areas of interest supplemental to their studies, or leisure—into their schedules. Audiobooks have become a way for people to “keep up on the books they want to read while they’re going about their day – be it while they’re commuting, or doing housework, or exercising, or winding down for bed” [11]. The data confirms this assertion: according to a 2020 study, 43% of adult audiobook listeners in the U.S. listened most often in the car; 73.4% of women and 66.1% of men reported multi-tasking while listening to audiobooks [2].

In this study, we provided students and faculty in health sciences programs with access to nonfiction, digital audiobooks related, but supplemental to, their fields of study through OverDrive’s Libby app as a pilot project. We tracked usage and cost of content areas and specific titles to help identify interest in audiobooks in our libraries and how to grow our collection in the future.

## CASE PRESENTATION

USF Health Libraries primarily serve the four colleges that comprise USF Health: medicine, nursing, pharmacy, and public health. The health libraries operate separately from the USF Libraries with our own dedicated leadership and budget, reporting directly to USF Health to prioritize the needs of the approximately 6,000 students under the USF Health umbrella.

In March 2024, we were inspired by a presentation at the Florida Health Sciences Library Association annual meeting about a hospital library using OverDrive’s Libby app to provide audiobook access to their patrons [12]. We decided to pilot an audiobook collection through OverDrive for our library population because audiobooks would be a new format not previously offered. Additionally, the Libby mobile app is easy to use and many patrons may already be familiar with it through the public library. Audiobooks could be a way for us to provide students and faculty with nonfiction content supplemental to the curricula. Finally, we know our health sciences students are busy, so we anticipated that audiobooks might be a way for students to consume content while doing other things.

### Vendor Options

While some competition exists for OverDrive in providing audiobooks to libraries, OverDrive is the “largest commercial provider” of such content [13]. Hoopla, while second to OverDrive in providing electronic and audiobook content to public libraries in the United States, does not seem to have much purchase among academic libraries, though some may provide access through statewide library cooperatives that also serve public libraries, such as UNC Pembroke through NC LIVE [14]. Additionally, Baker & Taylor’s Boundless product, which provided digital media options for libraries, is currently winding down operations along with its parent company [15]. While this closure was not yet announced when we began the project, Boundless was not well known to us. In all, OverDrive was the most robust and simplest choice for our project.

However, an early consideration revolved around whether OverDrive would provide the specialized content we need as an academic, health sciences library. We met with the vendor, who gave us trial access to their Marketplace platform. From there, we were able to search the available title options. We identified that the options provided met our collection development standards and our focus on nonfiction audiobooks related to the health sciences for this project.

Our next step was to investigate the several pricing models offered by the vendor and determine which best met our needs. The “one copy / one user” (OC/OU) model is a purchase model where the library owns the content, but, as the name suggests, each copy is only available to one user at a time. Users may therefore need to join waiting lists for popular titles, or the library would need to purchase multiple copies to meet demand. Another concern with this pricing model for our pilot program was that it required a large up-front expense with no guarantee that the content would be used.

The second pricing model we explored was the “cost per circ” (CPC) model. With CPC, the library selects an unlimited number of titles to add to their collection and then is charged fees as users borrow the titles. The “per circ” cost is a fraction of the cost to purchase a title outright and comes with the advantage of allowing multiple users to access a title simultaneously, with the library charged for each circulation of that title. Two other advantages with CPC for our purposes were that it would allow us to offer a wide selection of titles to our patrons and the library would only pay for the titles that patrons actually use. One disadvantage, however, is that fewer titles are available as CPC than in the OC/OU model.

We decided to use the CPC model as our primary pricing model, though we have purchased some titles through OC/OU when they weren’t available through CPC.

Regardless of pricing model, OverDrive requires a “content credit” deposit from which funds are deducted as purchases are made or titles are circulated. We started with the minimum content credit amount, which has been more than enough for the amount of circulation we’ve seen from our patrons.

### Selecting Titles

The overall focus for the pilot program was nonfiction health sciences audiobooks. Though OverDrive provides access to a wide variety of e-books through the Libby app, we decided to use the platform for audiobooks only. One reason was because with the many disparate platforms and sources the library already provides for e-books, paired with the self-contained nature of the Libby app, we were concerned patrons would search for specific e-books on Libby and assume that no result in Libby meant the library could not provide the book. We advertised the Libby app as a source for audiobooks and, because it is our only audiobook platform, patrons can safely assume that no result in Libby means the audiobook title is not available through the library.

We further narrowed our focus to include CPC titles that fell into one of four content areas: popular nonfiction, first person experiences, history of healthcare, and study helps. We later added an additional area: wellness. Popular nonfiction encompasses titles about health and medicine written for general audiences. First person experiences include memoir-type books giving the perspective of a health practitioner or patient. History of healthcare includes works detailing medicine throughout history. Study helps incorporate reference, review, and test prep materials. Finally, wellness encompasses titles related to self-care, stress management, and personal well-being.

These content areas were chosen because they closely align with the mission of the library of providing state-of-the-art biomedical information while also providing options that support student development and learning supplemental to the curricula. We purposely excluded most consumer health titles, such as those touting diet and exercise plans, because of the difficulty verifying the credentials of the authors and the trustworthiness of the information.

The research and education unit librarians assisted in selecting titles: they were given access to Marketplace to search the available titles and created “carts” of suggestions for the content management staff to evaluate, largely to ensure the titles were available in the CPC pricing model. We did add a limited number of titles that did not fall into any of the focus categories only if the research and education unit librarians recommended them.

Ultimately, we launched the pilot with 530 titles available for our patrons to borrow.

### Setting Up the Platform

With titles selected, the next step was to set up the Libby platform and customize it to our library. We activated automated lists or created curated lists for each of the content areas. These lists flow into the libraries’ Libby landing page, where the covers of select titles appear in visual carousels and thumbnails to encourage browsing and check out (see Figures 1 and 2).

**Figure 1:**
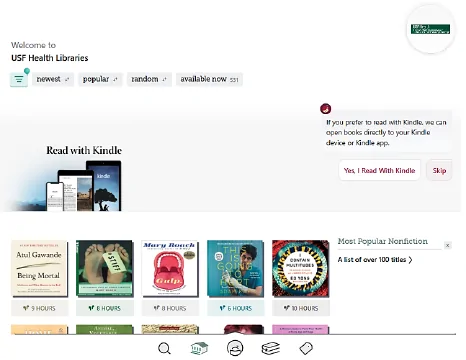
Browser view of USF Health Libraries Libby landing page.

**Figure 2:**
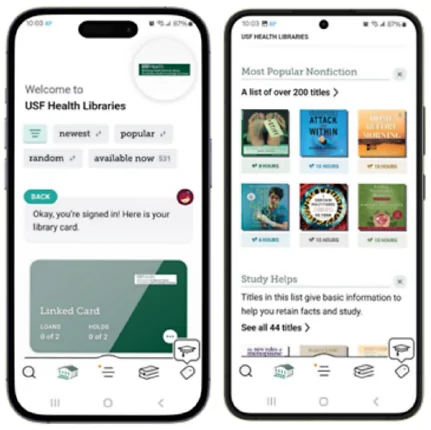
Mobile view of USF Health Libraries Libby landing page.

We also customized rules and policies such as loan limits and lending periods. Because of the experimental nature of the pilot, we viewed our initial choices for these settings as starting points, with the intention to adjust as needed as we got a better understanding of our patrons’ needs and the rate of spending. We started by limiting borrowers to two checkouts at a time and three total per month.

We set the default lending period to 21 days, though individual users can choose from a drop-down menu giving them the options of 7, 14, or 21 days each time they check out a title. Because we were relying on the CPC pricing model, we determined that setting the default lending period to the longest available might save the library money because it would make it less likely that a user would need to check out the same book twice due to running out of time.

### Cataloging and Access

Though Libby is a stand-alone mobile app and we anticipated that most users would access our libraries’ Libby content through the app, we also wanted to catalog the individual audiobook titles so that they would be discoverable through our libraries’ catalog and discovery layer.

OverDrive offers several ways to acquire MARC records, including both paid and free options with different levels of full or brief records. We chose the free MARC Express record option provided by OverDrive because the records were full enough for our needs. Institution-specific links to the audiobooks were provided in the 856 MARC field. This made it easy for our cataloger to download the MARC records directly from the OverDrive Marketplace site and batch upload them into our integrated library system.

To provide additional access points to the audiobooks, we also added Libby to our libraries’ A - Z database list.

### Marketing and Launch

We officially launched Libby audiobooks to our library community on August 1, 2024, ahead of the fall semester. We saw our first checkout later that afternoon.

Our marketing strategy revolved around advertising Libby audiobooks on as many university communication channels as possible. We promoted the service in the libraries’ monthly “Library Letter” email newsletter, created digital and physical flyers to post on bulletin boards and digital signage around campus, and encouraged our research and education librarians to mention Libby audiobooks in their classes and presentations.

We customized colors, graphics, and information on the standard marketing materials provided by OverDrive to ensure they reflected our branding and focus.

Soon after we launched the collection, a web and digital content developer joined our staff and has since helped us with refreshed marketing content and avenues for the Libby audiobook collection such as social media marketing and updated flyers and signage, including table tents and stall signs.

We utilized promotion campaigns targeted for Libby audiobooks approximately every other month in the first year of access, in November 2024, January 2025, March 2025, April 2025, May 2025, and July 2025.

## RESULTS

In the first year (August 2024 through July 2025) of offering audiobooks to our patrons, we saw 123 total checkouts of 64 titles across 74 unique users. Of our initial content credit, we spent $351.84 US on CPC checkouts.

### Usage

We saw the greatest number of checkouts in the popular nonfiction content area, with 45 total checkouts across 24 titles. See Figure 3 for a breakdown of checkouts across all content areas. The most frequently checked out popular nonfiction title was *Well: What We Need to Talk About When We Talk About Health* by Sandro Galea with 12 unique borrowers. This was also the title with the greatest number of checkouts overall during the first year.

**Figure 3:**
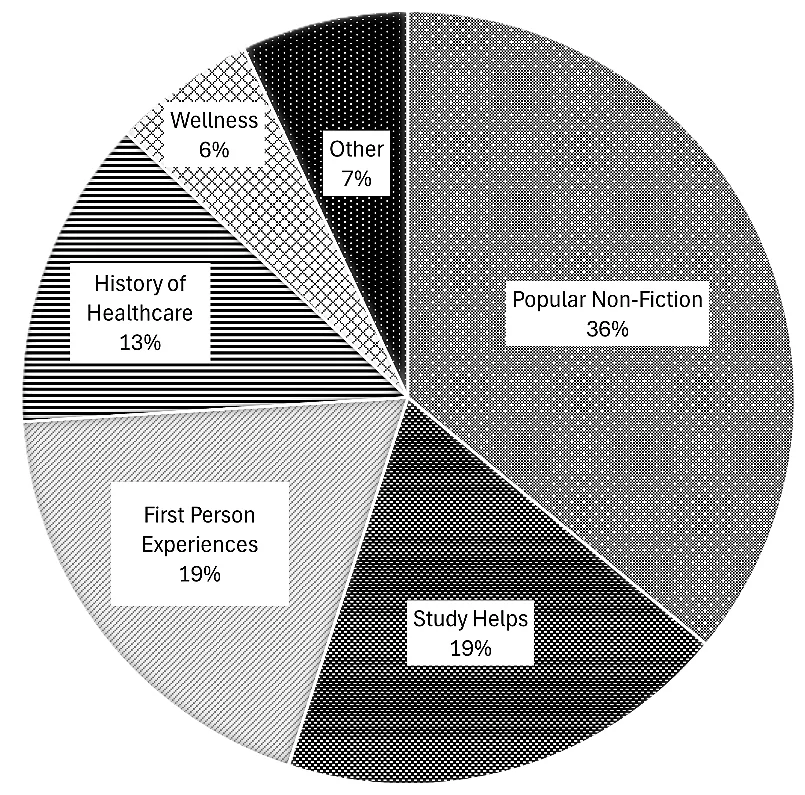
Libby audiobook checkouts per content area in the first year of access.

Two content areas tied for the second greatest number of checkouts: study helps and first-person experiences, with 23 checkouts across 12 titles each. The study help title checked out most often was *The New Rules of Menopause: A Mayo Clinic Guide to Perimenopause and Beyond* by Stephanie Faubion with 9 unique borrows. The first-person experiences title checked out most often was *This is Going to Hurt: Secret Diaries of a Young Doctor* by Adam Kay with 8 unique borrows.

“Other” refers to titles included in our collection, but that don’t easily fit into one of the primary categories. This includes six checkouts of a title we added to support a library-hosted book club and one checkout each for three titles related to leadership or education that our research and education librarians recommended but didn’t fall into one of the focus categories.

### Cost

We started August 2024 with $3,000 in content credit. We spent $351.84 of that deposit on the monthly CPC charges; an average of $29.32 per month. We spent an additional $430.80 on OC/OU e-book and audiobook purchases to support the library-hosted book club that met once per semester. Some titles the book club chose to discuss were not available under the CPC model or were not available as e-books through other platforms the libraries were already using. In all, we purchased eight e-book copies and one audiobook copy of three book club titles.

## DISCUSSION

Almost two-thirds of the titles used were checked out just once during the year, indicating that users were accessing a variety of titles from the available collection, even though only about 12% of the total available titles were checked out at all. These data confirm the decision to use the CPC pricing model to provide a wide selection of titles and only pay for what is used.

We finished the first year with more than $2,000 in content credit remaining and were then required to add more to that deposit amount for year 2 with OverDrive, so we added the minimum amount required.

On the one hand, it is positive that we did not exceed our budget, which was a plausible scenario due to the unpredictable nature of the CPC model. However, we finished the year with money still available to spend, meaning that the service was not used to capacity. We expect usage, and therefore cost, of the service will continue to increase as more patrons are made aware of it. So far in year 2, the monthly average spend for CPC checkouts is slightly higher than in year 1, at $30.87 per month based on the first six months of year 2 data.

The specific focus of our collection—nonfiction audiobooks related to health and medicine—versus the overall breadth of formats and content available through Libby limits the spending capacity of our project. Because of this, OverDrive’s requirement to add content credit each year could be a future limitation for us.

One limitation is that we have not yet incorporated feedback directly from patrons, though subject librarians participated in selecting the titles in our collection. In retrospect, finding a way to incorporate input from students and faculty in selecting titles would have been beneficial. When we selected the titles for the initial collection, we weren’t sure which areas would have the most demand, so we made broad selections. Now that we have some checkout data, we can be more targeted in the titles or collecting areas we choose going forward. Our next steps include gaining insight directly from our libraries’ Libby audiobook users to understand how their preferences and levels of satisfaction might shape the program going forward.

Another lesson learned was that we need to develop a process for refreshing the content available in the CPC audiobook collection. For one, titles may leave OverDrive or the CPC program without notice and therefore be removed from our collection unexpectedly. We need to be aware of these changes so that we can remove the title from our catalog manually. Additionally, as new titles are published or become available that meet our collection goals, we want to be able to make them available.

Through this project, we discovered that OverDrive’s title library includes sufficient offerings for academic health sciences libraries to create targeted nonfiction audiobook collections. The consistent and varied usage of the audiobook collection at USF Health Libraries in the first year indicates that it is benefitting some students or faculty members. The low average cost associated with the program thus far means that it is sustainable, though we understand that if interest and usage increases, so will the cost.

In all, this project directly supports one of our library’s strategic pillars: strengthening library collections. It does so in a way that offers supplemental health and medicine content in a modern format popular among the demographics of our students.

## Data Availability

Due to privacy concerns, circulation data are not available. Aggregate data related to circulation is available in the Appendix.

## SUPPLEMENTAL FILES

**Appendix A:** Sound Solutions Aggregate Data
