# Supplementary material for: Sound solutions: expanding access through digital audiobooks in an academic health sciences library
Source: J Med Libr Assoc. 2026 Oct 14;114(4):439–44. doi: 10.5195/jmla.2026.2425 (PMC13670221; doi:10.5195/jmla.2026.2425)
Supplement: Supplementary file 1 [file jmla-114-4-439-s01.docx]

**APPENDIX**

**Libby Audiobook Titles with Checkouts During Year 1 of the Project**

*Denotes a title discussed during a library-sponsored book club.300

***First Person Experiences***

| **Audiobook Title** | **Number of Checkouts (August 2024-July 2025)** |
| --- | --- |
| This Is Going to Hurt: Secret Diaries of a Young Doctor (unabridged) | 8 |
| Better: A Surgeon's Notes on Performance (unabridged) | 4 |
| Committed: Dispatches from a Psychiatrist in Training (unabridged) | 2 |
| The Neuroscientist Who Lost Her Mind: My Tale of Madness and Recovery (unabridged) | 1 |
| On Death and Dying: What the Dying Have to Teach Doctors, Nurses, Clergy and their Own Families (abridged) | 1 |
| Love, Medicine and Miracles: Lessons Learned about Self-Healing from a Surgeon's Experience with Exceptional Patients (unabridged) | 1 |
| Island Practice: Cobblestone Rash, Underground Tom, and Other Adventures of a Nantucket Doctor (unabridged) | 1 |
| Holiday SOS: The Life-Saving Adventures of a Travelling Doctor (unabridged) | 1 |
| Do No Harm: Stories of Life, Death, and Brain Surgery (unabridged) | 1 |
| Code Gray: Death, Life, and Uncertainty in the ER (unabridged) | 1 |
| Animal, Vegetable, Miracle: A Year of Food Life (unabridged) | 1 |
| All Bleeding Stops: Life and Death in the Trauma Unit (unabridged) | 1 |

***History of Health Care***

| **Audiobook Title** | **Number of Checkouts (August 2024-July 2025)** |
| --- | --- |
| The Facemaker: A Visionary Surgeon's Battle to Mend the Disfigured Soldiers of World War I (unabridged)* | 8 |
| The Good Nurse: A True Story of Medicine, Madness, and Murder (unabridged) | 2 |
| Plagues upon the Earth: Disease and the Course of Human History (unabridged) | 2 |
| Women in White Coats: How the First Women Doctors Changed the World of Medicine (unabridged) | 1 |
| The Cure for Women: Dr. Mary Putnam Jacobi and the Challenge to Victorian Medicine That Changed Women's Lives Forever (unabridged) | 1 |
| Quackery: A Brief History of the Worst Ways to Cure Everything (unabridged) | 1 |
| Moonshot: Inside Pfizer's Nine-Month Race to Make the Impossible Possible (unabridged) | 1 |

***Other***

| **Audiobook Title** | **Number of Checkouts (August 2024-July 2025)** |
| --- | --- |
| The Heaven & Earth Grocery Store: A Novel (unabridged)* | 6 |
| Teach Yourself How to Learn: Strategies You Can Use to Ace Any Course at Any Level (unabridged) | 1 |
| No Longer Welcome: The Epidemic of Expulsion from Early Childhood Education (unabridged) | 1 |
| Human-Centered Leadership in Healthcare (unabridged) | 1 |

***Popular Nonfiction***

| **Audiobook Title** | **Number of Checkouts (August 2024-July 2025)** |
| --- | --- |
| Well: What We Need to Talk About When We Talk About Health (unabridged) | 12 |
| I Contain Multitudes: The Microbes Within Us and a Grander View of Life (unabridged) | 3 |
| Being Mortal: Medicine and What Matters in the End (unabridged) | 2 |
| Black like Me (unabridged) | 2 |
| Gulp: Adventures on the Alimentary Canal (unabridged) | 2 |
| Mother Brain: How Neuroscience Is Rewriting the Story of Parenthood (unabridged) | 2 |
| Stiff: The Curious Lives of Human Cadavers (unabridged) | 2 |
| The Burnout Epidemic: The Rise of Chronic Stress and How We Can Fix It (unabridged) | 2 |
| The Secrets of Consciousness (unabridged) | 2 |
| We're Overdosed (unabridged) | 2 |
| Attack from Within: How Disinformation Is Sabotaging America (unabridged) | 1 |
| Deep Medicine: How Artificial Intelligence Can Make Healthcare Human Again (unabridged) | 1 |
| Enviromedics: The Impact of Climate Change on Human Health (unabridged) | 1 |
| Health Disparities in the United States: Social Class, Race, Ethnicity, and the Social Determinants of Health (unabridged) | 1 |
| Infectious Madness: The Surprising Science of How We "Catch" Mental Illness (unabridged) | 1 |
| Sweat: Uncovering Your Body's Hidden Superpower (unabridged) | 1 |
| The Death Gap: How Inequality Kills (unabridged) | 1 |
| The Drug Hunters: The Improbable Quest to Discover New Medicines (unabridged) | 1 |
| The Nocebo Effect: When Words Make You Sick (unabridged) | 1 |
| The Nocturnal Brain: Nightmares, Neuroscience, and the Secret World of Sleep (unabridged) | 1 |
| The Remarkable Life of the Skin: An Intimate Journey Across Our Largest Organ (unabridged) | 1 |
| The Upside of Aging: How Long Life Is Changing the World of Health, Work, Innovation, Policy, and Purpose (unabridged) | 1 |
| Welcome to Your World: How the Built Environment Shapes Our Lives (unabridged) | 1 |
| Your Brain, Explained: What Neuroscience Reveals About Your Brain and its Quirks (unabridged) | 1 |

***Study Helps***

| **Audiobook Title** | **Number of Checkouts (August 2024-July 2025)** |
| --- | --- |
| The New Rules of Menopause: A Mayo Clinic guide to perimenopause and beyond (unabridged) | 9 |
| NCLEX-RN Exam Prep 2024-2025: 500 NCLEX-RN Test Prep Questions and Answers with Explanations (unabridged) | 3 |
| NCLEX Review Book: Conquer the National Council Licensure Examination (NCLEX) on Your First Try \| Unleash an Extensive Range of Practice Tests \| Uncover Proven Tactics \| Fun and Interactive Learning Sessions \| Streamlined Path to Certification Victory! (unabridged) | 2 |
| The NCLEX-RN Exam Study Guide: Premium Edition: Proven Methods to Pass the NCLEX-RN Examination with Confidence – Extensive Next Generation NCLEX (NGN) Practice Test Questions with Answers (unabridged) | 1 |
| The Brain: A Very Short Introduction (unabridged) | 1 |
| Next Generation NCLEX-RN Exam Prep: Your Essential Guide to Mastering the National Council Licensure Examination for Registered Nurses \| Over 200 Thoroughly Explained Q&A \| Triumph on Your Initial Try! (unabridged) | 1 |
| Medical Terminology: Master Your Medical Vocabulary by Learning to Pronounce, Understand and Memorize over 2000 of the Most Commonly Used Medical Terms (unabridged) | 1 |
| Medical Coding and Billing--The Comprehensive Guide (unabridged) | 1 |
| Lab Values: An Easy Guide to Learn Everything You Need to Know About Laboratory Medicine and Its Relevance in Diagnosing Disease (unabridged) | 1 |
| Epidemiology For Dummies (unabridged) | 1 |
| Crunch Time Review for Pathophysiology (unabridged) | 1 |
| 1000 CWOCN Exam Practice Questions: Includes Detailed Answers with Explanations: Your Comprehensive Guide to Success in Wound, Ostomy, and Continence Nursing Certification (unabridged) | 1 |

***Wellness***

| **Audiobook Title** | **Number of Checkouts (August 2024-July 2025)** |
| --- | --- |
| The Power of the Gut-Brain Connection: How to Leverage the Gut-Brain Axis to Improve Your Physical, Mental and Emotional Well-Being (unabridged) | 2 |
| Reinvent the Wheel: How Top Leaders Leverage Well-Being for Success (unabridged) | 2 |
| The Self-Care Prescription: Powerful Solutions to Manage Stress, Reduce Anxiety & Increase Well-Being (unabridged) | 1 |
| Stress Management Tools: Coping With Stress for Mental and Physical Health and Longevity (unabridged) | 1 |
| Hello Sleep: The Science and Art of Overcoming Insomnia Without Medications (unabridged) | 1 |
